# From 2D to 3D: Promising Advances in Imaging Lung Structure

**DOI:** 10.3389/fmed.2020.00343

**Published:** 2020-07-16

**Authors:** Timothy Klouda, David Condon, Yuan Hao, Wen Tian, Maria Lvova, Ananya Chakraborty, Mark R. Nicolls, Xiaobo Zhou, Benjamin A. Raby, Ke Yuan

**Affiliations:** ^1^Divisions of Pulmonary Medicine, Boston Children's Hospital, Boston, MA, United States; ^2^Division of Pulmonary, Allery and Critical Care Medicine, Stanford University, Stanford, CA, United States; ^3^VA Palo Alto Health Care System, Department of Medicine, Stanford University, Stanford, CA, United States; ^4^Division of Pulmonary and Critical Care Medicine, Channing Division of Network Medicine, Brigham and Women's Hospital, Harvard Medical School, Boston, MA, United States

**Keywords:** lung structure, vibratome, precision cut lung slices, optical clearing, confocal, STED

## Abstract

The delicate structure of murine lungs poses many challenges for acquiring high-quality images that truly represent the living lung. Here, we describe several optimized procedures for obtaining and imaging murine lung tissue. Compared to traditional paraffin cross-section and optimal cutting temperature (OCT), agarose-inflated vibratome sections (aka precision-cut lung slices), combines comparable structural preservation with experimental flexibility. In particular, we discuss an optimized procedure to precision-cut lung slices that can be used to visualize three-dimensional cell-cell interactions beyond the limitations of two-dimensional imaging. Super-resolution microscopy can then be used to reveal the fine structure of lung tissue's cellular bodies and processes that regular confocal cannot. Lastly, we evaluate the entire lung vasculature with clearing technology that allows imaging of the entire volume of the lung without sectioning. In this manuscript, we combine the above procedures to create a novel and evolutionary method to study cell behavior *ex vivo*, trace and reconstruct pulmonary vasculature, address fundamental questions relevant to a wide variety of vascular disorders, and perceive implications to better imaging clinical tissue.

## Introduction

High resolution imaging of intact tissue is vital to understanding the anatomy and physiology underlying human disease. Especially in the lung, where cells and tissues interact with the different environmental and internal stimuli, robust visualization of tissue throughout the intact lung helps identify the patterns and characteristics of diseases from asthma to fibrosis to COPD (1).

Detailed imaging of murine lung tissue that preserves the structure and cellular components helps advance the knowledge and understanding of diseases so therapeutic approaches can be applied to human subjects. The ability to label protein, RNA, and other biological compounds with a high signal-to-noise ratio while maintaining physiological structure broadens the understanding of pathology and can demonstrate the spatial approximation of different biological compounds and cell types *in situ*.

Despite sophisticated advancements in imaging technology, acquiring high-quality, clinically relevant data remains challenging. The inherent difficulties in lung tissue preparation are the technical bottleneck. Sub-optimally prepared lung tissue can lead to inaccurate alveolar architecture, light scattering and difficulties with fluorescent staining, which may potentially produce an inaccurate model.

The natural respiratory cycle is driven by negative intrathoracic pressure generated by the respiratory muscles (2). Without this pressure gradient, as when a lung is harvested, the delicate pulmonary tissue collapses and no longer resembles its physiological structure. Preparing lung tissue for imaging requires inflating the collapsed airway and perfusing the vasculature, which changes the dynamic lung into a static fixture, maintaining structural integrity and molecular components. Given the different configuration and composition in alveolar and endothelial tissue, along with the rate at which the airways and vasculature taper in the lung, distinct techniques must be used to prepare tissue as realistically as possible.

Numerous different strategies and techniques exist for preparing animal and human tissue for visualization (1, 3, 4). The standard of practice for pathologists and histologists is preparing tissue in thin slices for microscopic visualization. However, advances in biomedical photonics has allowed advanced viewing of lung tissue, improving structure, resolution and clarity of samples. Precision-cut lung slices (PCLS) provides the advantage of maintaining the three-dimensional architecture of the lung compared to thin slices, allowing for dynamic views similar to physiological conditions. Advances in microscopy, including stimulated mission depletion microscopy (STED), bypass the diffraction limit of light microscopy providing super resolution images. Clearing optical techniques, such as solvent-based clearing protocols, has significantly improved the viewing of these biological specimens by decreasing the amount of light scattering (5). Current techniques focus on optimizing tissue clarity, retaining anatomical structure, and maintaining cellular molecules for fluorescence staining.

The primary purpose of this manuscript is to describe a novel, evolutionary method to prepare thin-sectioned, thick-sectioned, and whole-organ murine lung tissue that combines OCT tissue clearing, PCLS and tissue clearing into one protocol. The optimized sample provides high-quality images of preserved structures that can be fluorescence-stained. A secondary purpose of this manuscript is to review commonly used tissue preparation methods, discussing the pros and cons of each strategy.

## Materials and Methods

Please refer to Table 1 for instruments and equipment, chemicals and reagents and solutions used during methods.

**Table 1:** Equipment, Chemicals and reagents, Instruments and solutions used in the materials and methods.

**Equipment**
• Cryostat slicer (HM525NX, ThermoFisher)
• Vibration slicer (Leica VT1000s, Nussloch, Germany)
• Confocal microscope (Zeiss 880 with Airyscan)
• Light Sheet microscope (LaVision)
• Facility Line Confocal/STED microscopy (Abberior Instruments America LLC)
**Chemicals and reagents**
• UltraPure Low Melting Point Agarose (2%, Invitrogen,16520050)
• Mouse-anti-mouse/human SMA-Cy3 (1:300, Sigma, C6198-.2ML)
• Rat-anti-mouse CD31 (1:100; BD-Pharmingen, 553370 or 550274)
• Rabbit-anti-mouse-RFP (1:100, Rockland, 600-401-379)
• Goat anti-rabbit STAR RED (1:100, Abberior Instruments GmbH, STRED)
**Instruments and solutions**
• Flushing buffer (1X PBS + 0.1% heparin)
• PLP perfusion buffer (0.075 M lysine, 0.37 M sodium phosphate pH 7.2, 2% formaldehyde, and 0.01 M NaIO_4_, a detailed receipt can be found at Cold Spring Harbor Protocols)
• Inflation solution (½ volume of 100% OCT mixed with ½ volume of 30% sucrose)
• Expansion solution (2% agarose in 1XPBS)
• Blocking buffer (5% serum (goat, determined by species of secondary antibodies), 0.3% Triton X100 in 1XPBS)
• Antibody dilution buffer (1% BSA, 0.3% Triton X-100 in 1XPBS)
• MPBS (Percentage of Methanol in 1XPBS)
• BABB solution (1 volume of Benzyl Alcohol mix with 2 volume of Benzyl Benzoate)

### Animals

Animals used were 10–12 weeks, 22–25 g, male C57BL6 or NG2-Cre-ER/ROSA26RtdTomato (NG2-tdT) mice, as previously published (6). The NG2-tdT mouse line exhibits selective pericyte labeling by tdTomato when B6.Cg-Tg (Cspg4-Cre/Esr1^*^) BAkik/J (NG2-Cre-ER) mice (https://www.jax.org/strain/008538) are crossed with with tdTomato Ai14 (https://www.jax.org/strain/007908). NG2-tdT mice were treated with 2 mg tamoxifen dissolved in corn oil (20 mg/ml) for two consecutive injections. After tamoxifen treatments, mice rested for 7 days before tissue harvest.

### Harvest, Cannulation, and Perfusion of Mouse Lung

**Figure 1:**
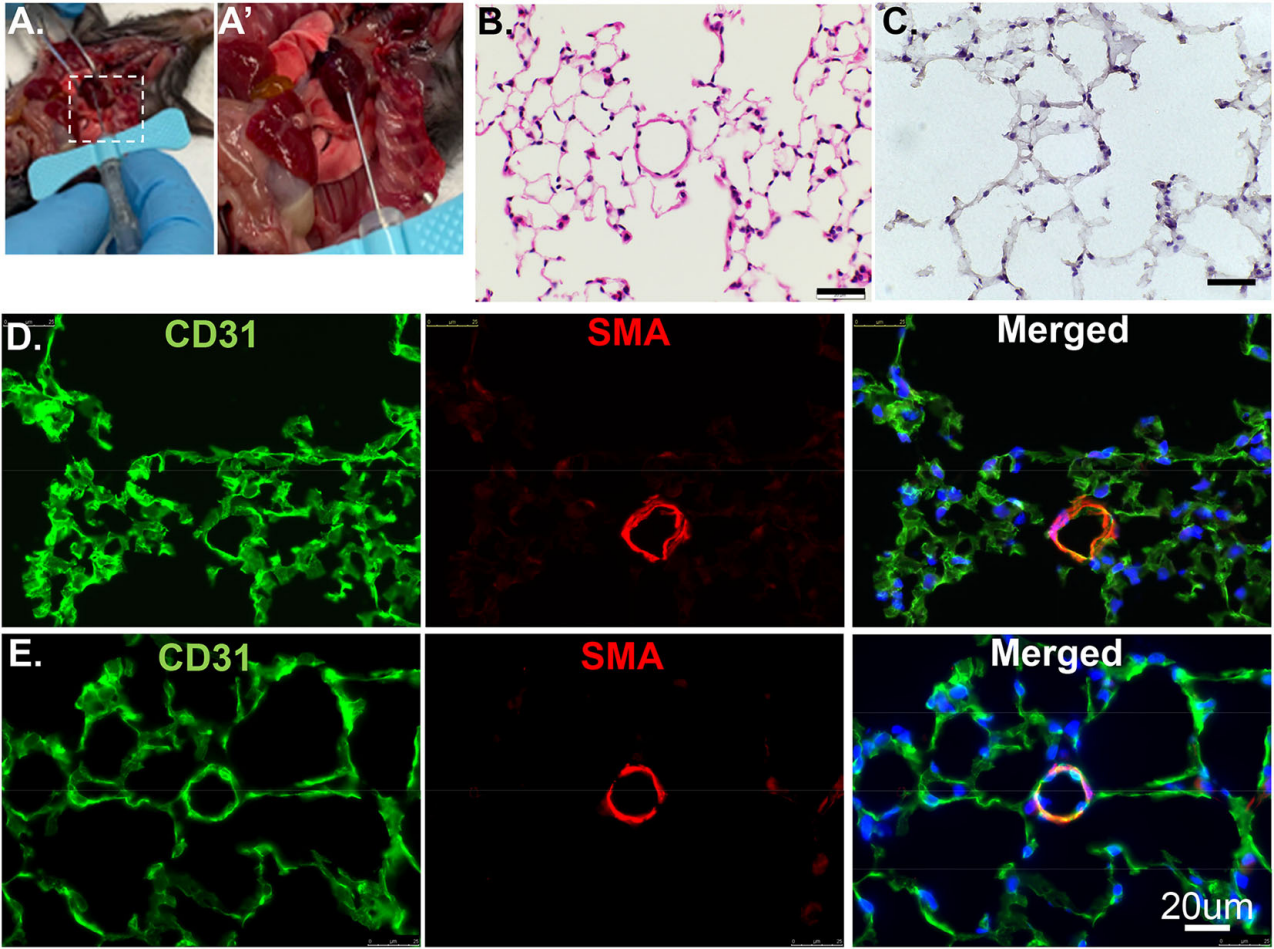
Cross section of lung tissues using H&E or IF. **(A)** The RV is cannulated with a 25G butterfly needle to facilitate switching solutions from flushing buffer to PLP for pulmonary vascular perfusion. **(A′)** Enlarged image of dashed rectangle in **A**. **(B)** Wildtype murine lung structure using H&E. Scale bar: 20 um. **(C)** Wildtype murine lung structure using H&E after inflation with 100% OCT. Scale bar: 20 um. **(D)** The same procedure as in C but applied IF staining. CD31: green, stained for endothelial; SMA: red, stained for smooth muscle layer; DAPI: blue, stained for nuclei. **(E)** PLP buffered murine lung described in manuscript providing improved structural integrity.

Anesthetize the animal with 3% isoflurane to ensure the lack of pain reflex, then sacrifice by neck isolation. Open the chest and create a small nick through the suprarenal abdominal aorta. Insert a 25–30 g butterfly needle^*^ (Figures 1A,A′) connected to a 30 cc syringe into the right ventricle. Flush with a 20 ml flushing buffer slowly but steadily until the fluid extravasating from the abdominal aorta is clear and colorless. ^*^Note: use of the butterfly needle prevents fluid leakage due to multiple cannulations.

(1) OCT Tissue Preparation

**Figure 2:**
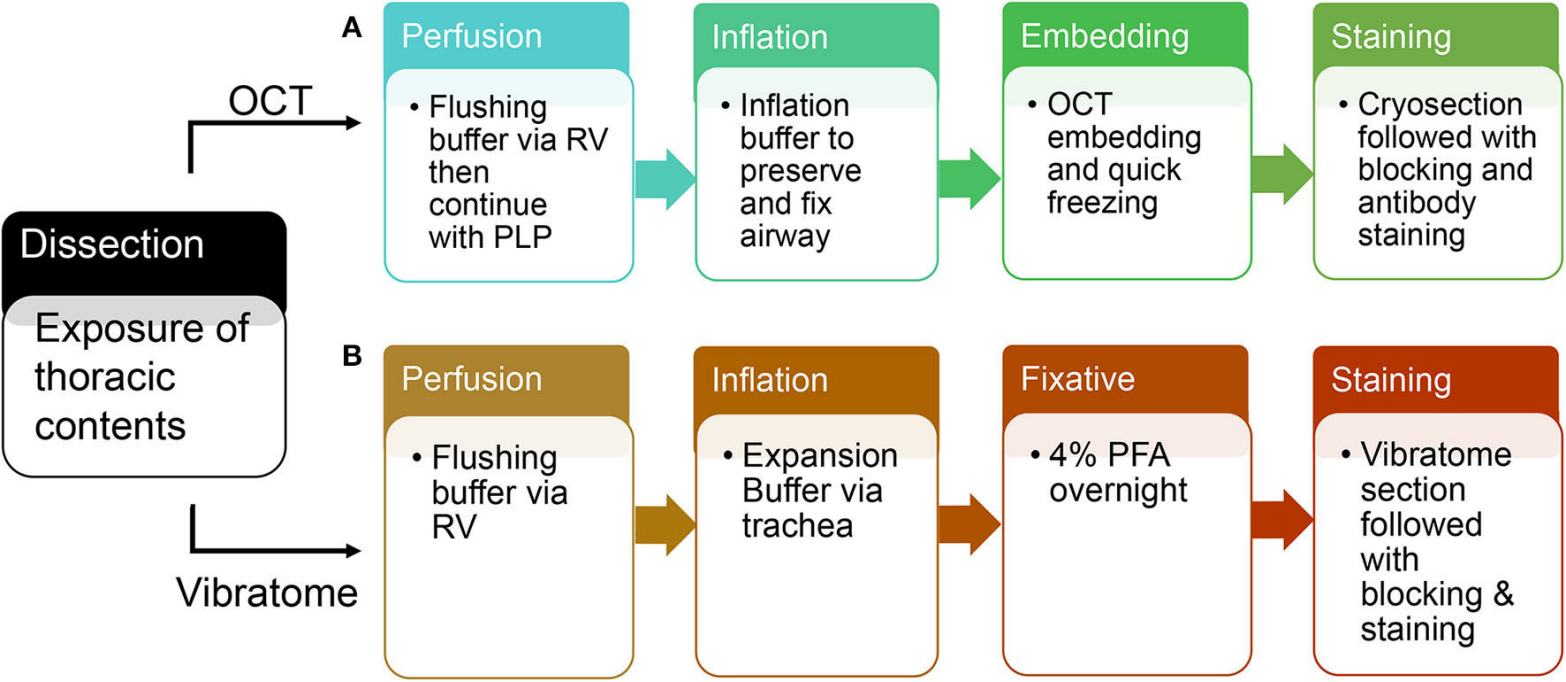
A schematic flow of OCT tissue preparation and vibratome tissue preparation. **(A)** An overview of steps in tissue preparation using OCT. **(B)** An overview of steps in tissue preparation using low melting agarose.

Switch syringe at infusion port of butterfly needle and flush with Periodate-Lysine-Paraformaldehyde (PLP) buffer. Cannulate trachea with 22 g blunt-ended needle and inflate with 2.5–4 ml OCT inflation solution. Immediately following inflation, separate and put each lobe in the tissue mold completely covered with OCT. Place the mold on a metal block and submerge in liquid nitrogen. After the OCT is completely frozen, store at −80°. Proceed with cryosectioning, immunolabeling, counterstaining, and imaging (each step is summarized in Figure 2).

(2) Vibratome

Cannulate trachea with 22 g blunt-ended needle and inflate with 2.5–4 ml 2% agarose solution. Incubate the dissected lungs in 4% paraformaldehyde solution overnight at 4° on an agitator. The next day, wash with 1XPBS at 4° and store for future use. Separate and section each lobe with Vibration Slicer at 300 um thickness. Block slices in serum overnight and incubate with primary/secondary antibodies in antibody dilution buffer, and lastly image with Zeiss confocal 880 (each step is summarized in Figure 2). Vibratome-sliced sections prepared for STED imaging will use the same protocol as Confocal, but incubate with STED designated secondary antibody (7), such as STAR Red.

(3) Tissue Clearing

Tissue clearing is effective with any thickness of tissue slice, including the whole lobe. Wash with PBS, then the following serial dilutions of MPBS: 25, 50, 75, 100% (dehydration) to 75, 50, 25%, PBS (rehydration), with an overnight incubation at each step. Incubation time will vary based on the thickness of the sample being prepared, with a minimum of 30 min recommended for thin samples (300 um) or overnight for whole lung lobes. The rest of the staining will be performed at 4°. Incubate in serum blocking buffer (the selected serum depends on the species of the secondary antibody, e.g., 5% Goat serum) overnight. Incubate primary antibodies in blocking buffer overnight. Wash slices in the blocking buffer at least three times. Incubate in secondary antibodies at least overnight. Wash slices in the blocking buffer at least three times. After a brief wash in PBS, dehydrate with serial dilutions of MPBS and stop at 100% methanol. Submerge tissue in BABB^*^ and gently agitate until the tissue is colorless. The organ must be as dehydrated as possible, as BABB will fail to clear tissue in the presence of water. Proceed with imaging with Confocal or Light-Sheet Microscopy. ^*^Note: Once combined, BABB is extremely volatile and toxic on skin contact.

## Results

Paraffin embedding is the traditional technique to preserve and evaluate lung structure. Paraffin sections are a valuable resource to reveal structural and matrix components details, such as procedures Movat, Trichrome, H&E staining (Figure 1B). However, paraffin embedding is suboptimal for immunofluorescence imaging. In this method, the lung is infused through airways with 3% formalin to preserve the structure and prevent artifacts that can preclude accurate histological evaluation. The fixative also cross-links certain proteins in and on the cells. Thus, antigen retrieval to “unmask” cross-linked antigens is often essential and required. Unfortunately, some antigens can be destroyed by the high-temperature processing used in “unmasking” and are no longer recognized by their designated antibodies. Sometimes, high antibody concentration throughout the tissue may cause non-specific binding as false-negative results.

Embedding in OCT (aka frozen sections) compound, a method with minimal antigen damage is much better for two-dimensional immunofluorescence (IF) imaging. OCT compound was developed specifically to facilitate tissue cryo-sectioning and permeability. Tissue preparation with OCT allows for faster start-to-finish tissue processing, thinner sections (10 um), with more consistent antigen penetration for immunolabeling when compared to paraffin preservation and sectioning.

Previously, we used PBS to flush blood from the harvested tissue, 3% formalin as a fixative, then inflated through airways with 100% OCT. After H&E and antibody staining, we found discontinuous layers of alveolar pneumocytes and transparent/matrix basement membrane, which failed to reflect the real structure (Figures 1C,D). To optimize this procedure, we used Periodate-Lysine-Paraformaldehyde (PLP) and inflation solution to preserve structural integrity. After a flushing buffer, PLP solution fixes the vasculature from the inside out while further clearing the tissue of blood. The inflation buffer optimizes the structural preservation of the alveolar/capillary interface even at the periphery and reduces autofluorescence (Figure 1E). We recommend performing the inflation step as quickly as possible to facilitate rapid coating with OCT and freezing. This step will exclude air bubbles and optimize the overall structure appearance.

**Figure 3:**
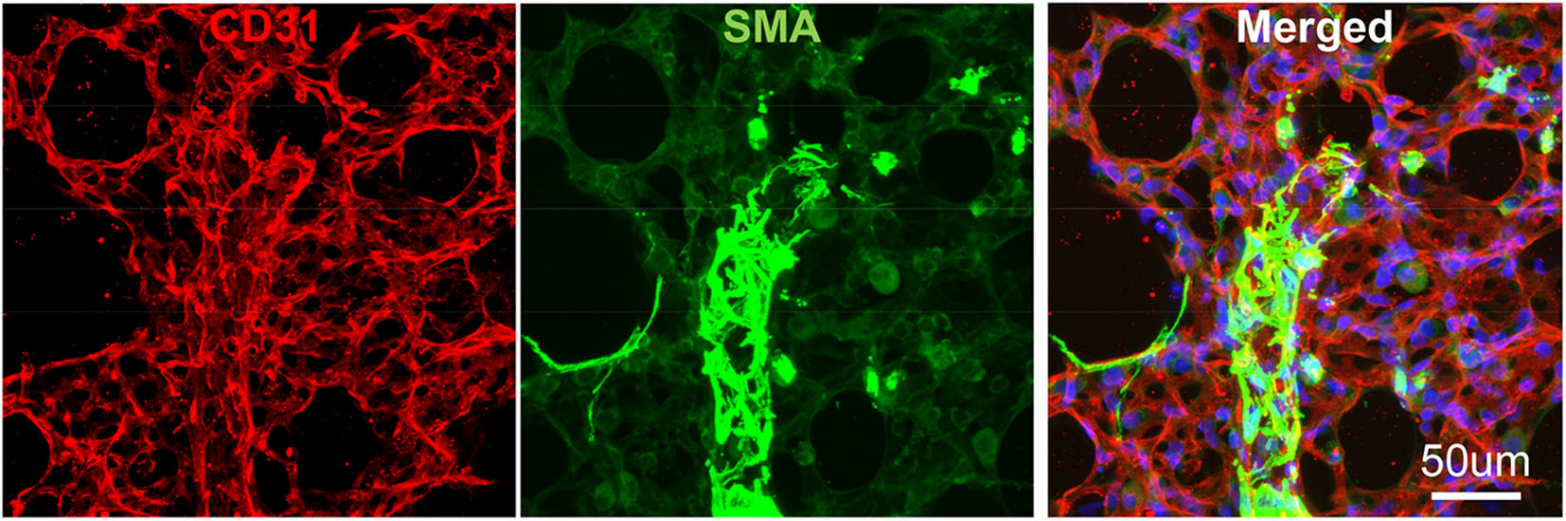
Vibratome section of lung tissues using IF. The wildtype murine lung after flushing was inflated with agarose and proceed with fixative. CD31: green, stained for endothelial; SMA: Red, stained for smooth muscle layer; DAPI: blue, stained for nuclei.

To avoid regional sampling bias when analyzing lung structure in a whole-mount context, we applied the expansion solution via trachea and sectioned into 300–600 um vibratome slices (the slice thickness can be determined by the working distance of confocal objective lens), or as known as Precision Cut Lung Slices (PCLS). These slices can be easily permeabilized and stained with antibodies CD31 for endothelium and SMA for smooth muscle. We then used confocal Z-stack/reconstitution to assess CD31/SMA-positive cell coverage on individual vessels (Figure 3). Quantification of our results can be achieved by counting the number of overlapped nuclei with or without positive antibody staining (8).

**Figure 4:**
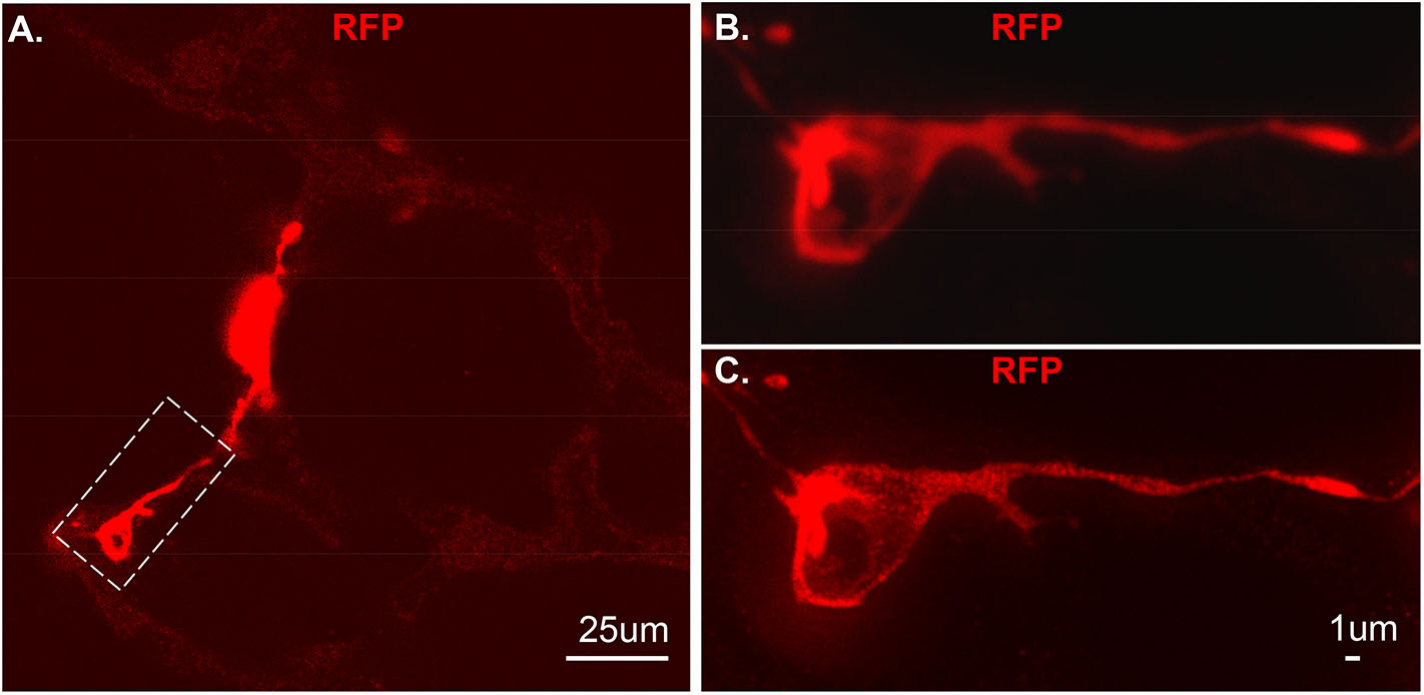
An individual tdT positive cell from vibratome sections. The section was stained using RFP and the secondary antibody used is anti-rabbit-STAR RED. **(A)** An overall view of NG2-tdT lung using STED. A RFP positive cell in dash rectangle was captured using Confocal **(B)** or captured using STED **(C)**. RFP: red fluorescence protein.

As proof of principle, we used a previous created a NG2-selective reporter mouse and showed that intraperitoneal tamoxifen selectively marks NG2 promoter-driven cells with red fluorescence in capillaries (6). The vibratome slices collected from this transgenic line was stained with RFP and imaged using Stimulated emission depletion (STED) microscopy (Figure 4A). Compared to the out-of-focus blur and an artifact seen by confocal (Figure 4B), STED allowed high resolution and distinction of single positive RFP on the cytoplasm at high spatial density (Figure 4C).

**Figure 5:**
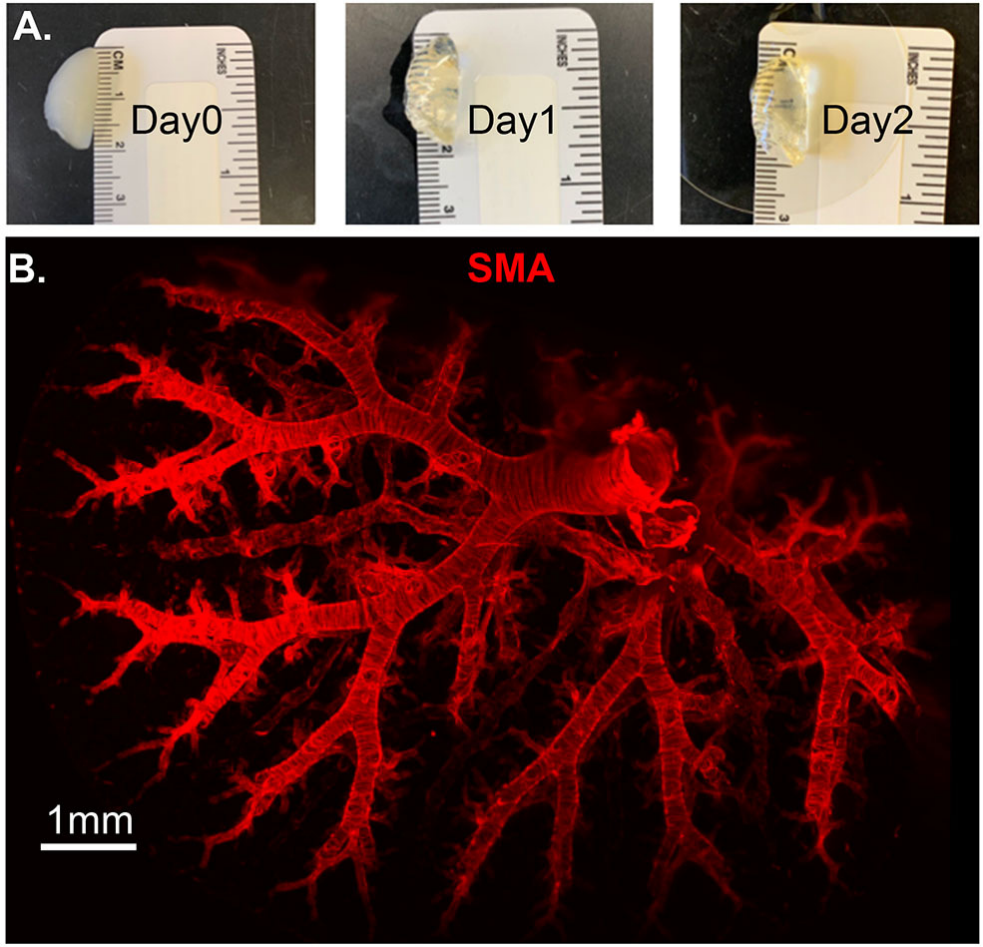
Lung structure staining by smooth muscle actin (SMA) staining. Wildtype mouse whole lung lobe was gradually and optically cleared (became transparent) in 2 days with organic solvent BABB **(A)** and imaged using 3D Light Sheet **(B)**.

Molecular and optical interrogation of large-sized biological tissue (>1 mm) can be achieved by combining optical tissue clearing and light-sheet microscopy (Figure 5). It enables a true representation of cell location in a whole organ level that allows the immunolabeling of proteins that are essential under normal and pathologic conditions. BABB clearing after systematic tissue dehydration, rehydration, labeling, and then second dehydration provides for a relatively fast protocol for this visualization. Positive SMA stain area quantification can be calculated by processing with the “Surface and Filament Tracer” tool of Imaris (Bitplane) (9).

## Discussion

In this manuscript, we outline new, enhanced methodologies to produce high-resolution images that preserve the structure and optimize experimental flexibility on thin-section, thick-sections, and whole organ images. The combination of tissue clearing preparation with lung inflation enables an unparalleled system-based visualization of biological pathways within the whole organ. Inflating the lung gives us a better perspective of distal vs. proximal structures and provides superior spatial detail compared to methods not used in combination.

The preparation of tissue microslices is a technique that uses a vibratome, or vibrating blade, to produce precision-cut organotypic slices. This allows for increased accuracy and reproducibility when generating precision-cut lung slices (PCLS) compared to alternate methods (10). Tissue slices are time- and cost-effective compared to other *in vivo* work (11, 12). PCLS has been used in human and animal studies focusing on lung anatomy, toxic exposures, infectious disease and immunological studies (13, 14). They can maintain the structural framework at both the tissue and cellular level, making them useful for studying anatomical abnormalities seen in diseases, such as asthma or COPD (15). This makes PCLS the ideal platform to study tissue-specificity and cancer cell selectivity of gene therapy vectors prior to *in vivo* trials (4).

However, PCLS does not come without limitations. PCLS displays a snapshot of the cells and molecules residing in lung tissue at the time of removal and does not capture the heterogeneity seen in some pathological processes. The PCLS model is also static, which means that any testing involving mechanical stress, such as barotrauma, is limited. Samples have a viability of 3–6 days, limiting the extend that PCLS can model *in vivo* situations (12). While a PCLS is an excellent model for physiologic and toxicologic studies, future investigation is needed to describe methods for improved viability incorporation of immune components to enhance PCLS.

Confocal imaging was patented in 1957 and aimed to overcome the limitations of traditional fluorescence microscopes when viewing thin-cut samples (16). It is superior to conventional microscopy by focusing light inside tissue and limiting the emitted light returned, allowing the specimen to be imaged at a single “point” at a time. This allows the reconstruction of high-resolution, high contrast three-dimensional images without the artifacts that limit conventical microscopy. However, confocal imaging is restricted by its prolonged scanning time, photobleaching secondary to long scan times, causing reduced signal-to-noise ratios, and the inability to simultaneously visualize multiple specimen layers (17).

Due to these limitations, techniques were developed which split fluorescence light into multiple planes. This method, or light sheet microscopy, significantly reduced scanning time and some of the limitations seen with confocal imaging. Light sheet microscopy has been employed to investigate inflammation, cancer, hemopoiesis and infection in lung and other tissues (18–21). It has also has been used to investigate the distribution of *Aspergillosis fumigatus* and identify the local inflammatory response secondary to pulmonary infections (22). However, this technique is limited by optical aberrations and a point-by-point scanning technique (17).

Stimulated emission depletion (STED) microscopy creates super-resolution images at a molecular level. This overcomes the diffraction limitations seen in confocal microscopy to improve resolution and provide nanoscale visualization of individually labeled molecules (23). This technique has been recently used in neuroscience to better visualize dendritic cells and understand their structure and function (24). STED has been applied to lung tissue by detecting filamentous human respiratory syncytial virus particles, providing a better understanding and mechanism of how the virus spreads cell to cell (e.g., microbiota activity) (25). STED microscopy can potentially aid in understanding the molecular mechanisms of receptors and their specific ligands in disease, such as pulmonary hypertension, fibrosis and malignancies (26, 27). An understanding of these molecules at their response can lead to the development of possible pharmaceutical therapies.

Tissue optical clearing is a chemical process aiming to improve light penetration throughout intact tissue, rendering them transparent and allowing fluorescent microscope imaging (28). Organic-solvent based clearing protocols, such as 3DISCO can achieve high levels of tissue transparency, allowing imaging of large samples, such as brain, tumors and embryos (5, 29). However, the inability to preserve fluorescent protein emission with solvent-based clearing techniques has led to a pursue with aqueous-based solutions. Current techniques include passive immersion, hyperhydration, or hydrogel embedding. While these techniques traditionally do not clear as well as solvent-based methods, they are easy to implement and useful in samples where a wide range of fluorescent dyes and proteins are required. CLARITY is a method for chemically transforming intact biological tissue into a hydrogel-tissue hybrid (3). This hybrid model is amendable to light and macromolecular labels while also retaining structure and biological molecules, such as proteins and nucleic acids. CLARITY has the additional benefit of being plastic-safe, making materials easier to select for processing and imaging safer on most institutional equipment due to lack of necessity for imaging while submerged within an organic solvent. The method has also been successfully applied to numerous animal and human brain models, with the potential to be applied to different, large organ systems. For lung tissue mainly, we found the solvent-based method is more stable and less time-consuming than CLARITY due to variable lipid and protein contents.

In summary, our study provides novel and insightful methods to improve and enhance experimental procedures to preserve pulmonary vasculature with minimal change from regional to a whole-organ large scale. Additionally, all presented methods can have a broader and more comprehensive application, such as assessment of lung pathology in clinical samples.

## Data Availability Statement

The raw data supporting the conclusions of this article will be made available by the authors, without undue reservation.

## Ethics Statement

The animal study was reviewed and approved by IACUC at Boston Children's Hospital and Stanford University.

## Author Contributions

KY: conception and design, acquisition of data, analysis and interpretation of data, drafted and revised the manuscript. TK and DC: analysis and interpretation of data, drafted and revised the manuscript. YH, WT, ML, AC, MN, XZ, and BR made substantial contributions to the acquisition of data, analysis and interpretation of data. All authors contributed to the article and approved the submitted version.

## Conflict of Interest

The authors declare that the research was conducted in the absence of any commercial or financial relationships that could be construed as a potential conflict of interest.
